# Dysregulation of lysophosphatidic acids in multiple sclerosis and autoimmune encephalomyelitis

**DOI:** 10.1186/s40478-017-0446-4

**Published:** 2017-06-02

**Authors:** K. Schmitz, R. Brunkhorst, N. de Bruin, C. A. Mayer, A. Häussler, N. Ferreiros, S. Schiffmann, M. J. Parnham, S. Tunaru, J. Chun, S. Offermanns, C. Foerch, K. Scholich, J. Vogt, S. Wicker, J. Lötsch, G. Geisslinger, I. Tegeder

**Affiliations:** 1 pharmazentrum frankfurt, Institute of Clinical Pharmacology, Goethe-University Hospital, Frankfurt am Main, Germany; 2 Department of Neurology, Goethe University Hospital, Frankfurt am Main, Germany; 3 Fraunhofer Institute of Molecular Biology and Applied Ecology - Project Group Translational Medicine and Pharmacology (IME-TMP), Frankfurt am Main, Germany; 4 Institute for Microscopic Anatomy and Neurobiology, University Medical Center, Johannes Gutenberg-University, Mainz, Germany; 5 Max Planck Institute for Heart and Lung Research, Bad Nauheim, Germany; 6 Sanford Burnham Prebys, Medical Discovery Center, La Jolla, CA USA; 7 Occupational Health Service, Goethe-University Hospital, Frankfurt am Main, Germany; 8 Institute of Clinical Pharmacology/ZAFES, Goethe-University Hospital, Frankfurt, Germany

**Keywords:** Lysophosphatidic acids, Multiple sclerosis, Autoimmune encephalomyelitis, *Lpar2*, Neuroinflammation, Spinal cord, T-cell homing

## Abstract

**Abstract:**

Bioactive lipids contribute to the pathophysiology of multiple sclerosis. Here, we show that lysophosphatidic acids (LPAs) are dysregulated in multiple sclerosis (MS) and are functionally relevant in this disease. LPAs and autotaxin, the major enzyme producing extracellular LPAs, were analyzed in serum and cerebrospinal fluid in a cross-sectional population of MS patients and were compared with respective data from mice in the experimental autoimmune encephalomyelitis (EAE) model, spontaneous EAE in TCR^1640^ mice, and EAE in *Lpar2* ^-/-^ mice. Serum LPAs were reduced in MS and EAE whereas spinal cord LPAs in TCR^1640^ mice increased during the ‘symptom-free’ intervals, i.e. on resolution of inflammation during recovery hence possibly pointing to positive effects of brain LPAs during remyelination as suggested in previous studies. Peripheral LPAs mildly re-raised during relapses but further dropped in refractory relapses. The peripheral loss led to a redistribution of immune cells from the spleen to the spinal cord, suggesting defects of lymphocyte homing. In support, LPAR2 positive T-cells were reduced in EAE and the disease was intensified in *Lpar2* deficient mice. Further, treatment with an LPAR2 agonist reduced clinical signs of relapsing-remitting EAE suggesting that the LPAR2 agonist partially compensated the endogenous loss of LPAs and implicating LPA signaling as a novel treatment approach.

**Graphical abstract:**

Graphical summary of lysophosphatidic signaling in multiple sclerosis
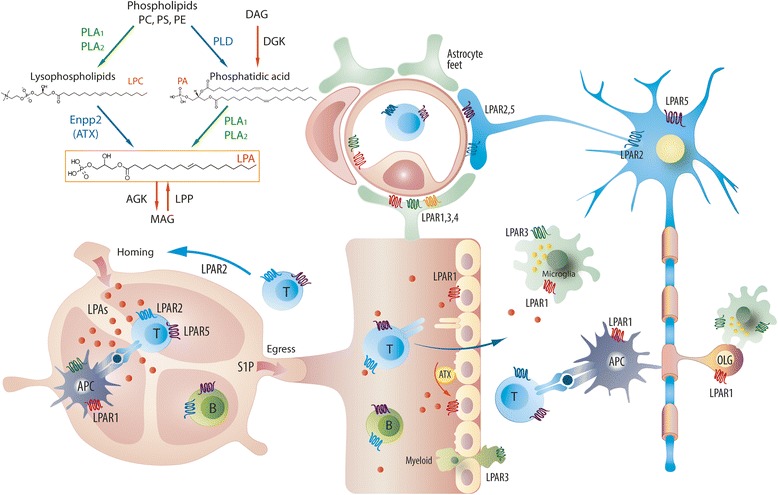

**Electronic supplementary material:**

The online version of this article (doi:10.1186/s40478-017-0446-4) contains supplementary material, which is available to authorized users.

## One sentence summary

Lysophosphatidic acids are reduced in patients with multiple sclerosis and in EAE mice, and the consequent loss of LPAR2 signaling in immune cells promotes the disease.

## Introduction

Lysophosphatidic acids (LPAs) are lipid-signaling molecules of different chain lengths and phosphorylation. Presently, 6 different LPAs can be detected in biological fluids or tissue extracts [37] and they signal through specific, functionally diverse G-protein coupled LPA receptors, LPAR1-5, and the atypical LPAR6 [6, 33]. LPARs are differentially expressed by various kinds of immune cells and may preferentially engage saturated or mono/poly unsaturated LPAs [4]. Signal transduction through LPARs regulates cell mobility and migration [25, 71], neuro - and angiogenesis [62], and platelet aggregation [43]. Their functions in the context of multiple sclerosis (MS) are presently unknown.

The discovery of the mechanisms for extracellular LPA generation has stimulated research of their function for innate and adaptive immunity and immune cell signaling. There are two major pathways for extracellular LPA production [37]: (i) by direct hydrolysis of a fatty acid moiety from membrane-derived phosphatidic acid [18] and (ii) by removal of the choline moiety from the abundant lysophosphatidylcholine (LPC) [56, 60]. The latter process is mediated by autotaxin (ATX), a secreted lysophospholipase D (lyso-PLD), which is a multidomain and multi-functional protein, with lyso-PLD and phosphodiesterase enzyme activities [40, 56, 67].

ATX is normally expressed by high endothelial cells of lymph node venules i.e. at the sites of immune cell transmigration [2]. It is also expressed by oligodendrocyte precursor cells [47] and activated astrocytes [47], and promotes the morphological maturation of glial cells [14] and myelination [19]. On stimulation, autotaxin binds to integrin of cell surfaces via its somatomedin-B like domain [21, 26], then utilizes LPC of the target cell membrane to produce LPAs in the close vicinity of the targeted LPA receptor, a mechanism that confers specificity. The ATX-mediated targeted release of LPAs may orchestrate LPA-effects in lymphoid organs and at the blood brain barrier and hence immune cell adhesion, invasion into or evasion from the brain [38].

Studies with small molecular autotaxin inhibitors have suggested that LPAs have pro-inflammatory functions [29, 50], but the outcome depends on the where and when of their production, the target receptor [44, 72], and the ability to maintain a balance between production and breakdown, the latter mediated by membrane bound lipid phosphate phosphatases (LPPs) [57]. The sphingsosine 1-phosphate (S1P) receptor antagonist fingolimod, which is one of the most effective relapse preventing drugs in relapsing remitting multiple sclerosis (RRMS) [10], has been reported to also be a slow acting autotaxin inhibitor [57] and protects against deregulation of signaling lipids at the blood brain barrier [61]. It is unknown to what extent this effect contributes to, or interferes with its efficacy in MS.

To gain further insight into the functions of the ATX/LPA axis for neuroimmunological diseases we used a translational approach. We analyzed LPAs and autotaxin in a cross sectional population of MS patients and in mice in the experimental autoimmune encephalomyelitis model (EAE), using immunization-evoked primary progressive and relapsing-remitting EAE, spontaneous EAE, and EAE in *Lpar2* deficient mice, the latter to assess functional implications of LPA signaling and LPAR-dependent immune cell redistribution. Finally, we assessed the therapeutic effects of an LPAR2 agonist.

## Materials and methods

### Patients with multiple sclerosis

Human samples and data were available from an observational cross-sectional investigation of 102 multiple sclerosis patients (31 men, 71 women, demographic data Table 1) consecutively recruited from outpatients and inpatients of the Department of Neurology of the Goethe University Hospital Frankfurt, Germany. Data and blood collection was part of the local bio-banking project (Neurological Department of the Goethe University, Frankfurt), adhered to the Declaration of Helsinki and was approved by the Ethics Committees of the Medical Faculty of the Goethe University. Cerebrospinal fluid samples were available from a previous study [48] including 20 MS patients and 10 control patients with other non-inflammatory neurologic diseases. For long-term time course analyses additional 15 patients were recruited and observed up to 3.5 years. Control samples were obtained from a cohort of 301 healthy subjects in total (118 men, 183 women, aged 18 – 57 years), enrolled in the Occupational Health Service at the University Hospital of Frankfurt, Germany. The local Ethics committee approved the enrollment and sample acquisition, and informed written consent from each participating subject was obtained. Venous blood samples were collected to serum tubes and centrifuged at 3,000 rpm for 10 min. Serum was frozen at −80 °C pending analysis.

**Table 1:** Demographic data of Multiple sclerosis patients and healthy control subjects

		Multiple sclerosis patients									Healthy controls		
		Male				Female							
		MS 1st course	RRMS stable	RRMS acute relapse	SPMS or PPMS	MS 1st course	RRMS stable	RRMS acute relapse	SPMS or PPMS	Total number (m/f)	Male	Female	Total
Number (all treatments)		8	16	4	3	12	39	18	2	102 (35/67)	118	183	301
Age (Years mean ± SD)		38.2 ± 7.5	35.5 ± 0.4	33.9 ± 7.0	43.6 ± 18.1	31.6 ± 9.1	34.3 ± 8.9	37.2 ± 10.9	49.3 ± 11.0	35.5 ± 9.7	25.0 ± 6.1	24.9 ± 6.1	25.0 ± 6.1
Years since diagnosis		0.1 ± 0.4	7.6 ± 6.1	2.1 ± 1.8	5.7 ± 4.7	0.8 ± 0.2	7.4 ± 5.9	6.7 ± 5.3	1.1 ± 1.3	5.6 ± 5.7			
Relapse prophylaxis Number of patients	No drug	6	3	1	1	11	6	10	1	39			
	Interferon	0	6	2	0	0	11	5	1	25			
	Fingolimod	0	3	0	1	0	5	1	0	10			
	Natalizumab	0	4	0	0	0	15	0	0	19			
	Other	2	0	1	1	1	2	2	0	9			

### Animals, drug treatments and kinetics

Female SJL/J mice (Envigo, Germany), aged 10–12 weeks at immunization were used for study of relapsing-remitting EAE and female TCR^1640^ mice for spontaneous RR-EAE [45]. Female C57BL6/J mice and LPAR2^−/−^ mice and LPAR2^+/+^ littermates were used for the study of primary progressive EAE. LPAR2^−/−^ mice have a mixed C57BL6/J x Sv129 background with about 25% Sv129. Mice were housed at 3–5 mice per cage at constant room temperature (21 ± 1 °C) under a regular 12 h light/dark schedule. Food and water were available *ad libitum*. Animals were treated orally via the drinking water with FTY720 (fingolimod) at 0.5 mg/kg/d starting 3 days after immunization for preventive treatment. Monoclonal anti-Itga4 antibody treatment (natalizumab) in SJL/J mice consisted in 3 intraperitoneal injections (1x200 μg, 2x 150 μg) on day 11, 14 and 19. The LPAR2 agonist GRI 977143 was administered perorally in cornflakes soaked with 10% sucrose/10% DMSO in water with 100 μg/mouse/d of the drug starting 3d after immunization. Control animals received the respective vehicle. Oral bioavailability (F) of the LPAR2 agonist and basic kinetics were assessed by LC-MS/MS analysis of plasma concentrations. Tmax and Cmax were directly taken from the plasma concentration time courses. AUCs were calculated according to the linear trapezoidal rule. The clearance was calculated as Cl = Dose*F/AUCs and the half-life, t1/2 = ln2/λ, where λ is the terminal decay constant.

The experiments adhered to the guidelines of the Committee for Research and Ethical Issues of the International Association for the Study of Pain (IASP) and to those of GV-SOLAS for animal welfare in science and the ARRIVE guidelines. They were approved by the local Ethics Committee for Animal Research (Darmstadt, Germany).

### EAE models

C57BL6/J and LPAR2 mice were immunized according to a standard protocol using the Hooke Kit™ MOG35-55/CFA emulsion PTX (EK-2110, Hooke Labs, St Lawrence, MA), which contains 200 μg myelin oligodendrocyte glycoprotein (MOG) 35–55 emulsified in 200 μl Complete Freund’s Adjuvant (CFA). The emulsion was injected subcutaneously at two sites followed by two intraperitoneal (i.p.) injections of 200 ng pertussis toxin (PTX) in phosphate buffered saline (PBS), the first 1–2 h after MOG35-55, and the second 24 h later. SJL mice were immunized using Hooke Kit™ PLP139-151/CFA emulsion PTX (EK-2120), which contains 200 μg myelin proteolipid protein (PLP) 139–151 in 200 μl CFA (Hooke Labs, US). Injections of the emulsion and PTX were done as described for C57BL6 mice.

TCR^1640^ transgenic mice carrying a T-cell receptor (TCR) specific for myelin oligodendrocyte glycoprotein (MOG) peptide 92–106 develop spontaneous EAE around the age of 3 months [45]. TCR^1640^ positive male mice were mated with wild type female SJL/J mice. TCR^1640^ positive litter were identified by FACS analysis of peripheral blood T-cells by identification of the TCRα8.3 and TCR-β4 chains using specific antibodies for Vα8.3 (B21.14) and Vβ4 (KT4) (Becton Dickinson) [45]. Female TCR^1640^ positive mice were observed for up to 6 months.

EAE scores were assessed daily to evaluate the severity and extent of motor function deficits. Score 0, normal motor functions; score 0.5, distal paralysis of the tail; score 1, complete tail paralysis; score 1.5, mild paresis of one or both hind legs; score 2, severe paresis of hind legs; score 2.5, complete paralysis of one hind leg; score 3, complete paralysis of both hind legs; score 3.5, complete paralysis of hind legs and paresis of one front leg. Serum and tissues were sampled after the second peak, 35–36 days after immunization.

Blood was sampled by puncture of the retrobulbar plexus or by cardiac puncture (final sample) into EDTA K^+^ microvettes for plasma or microvette® 200 Z-gel (Sarstedt) for serum samples. Blood was centrifuged at 3000 rpm for 10 min in a table centrifuge and plasma or serum samples were stored in standard Eppendorf caps at −80 °C until analysis. Tissue from specific brain regions and spinal cord and peripheral tissues (spleen, lymph nodes) were rapidly dissected and snap frozen on dry ice and stored at −80 °C.

### Analysis of lysophosphatidic acids

Quantitative LPA analysis was performed with liquid chromatography-electrospray ionization-tandem mass spectrometry (LC-ESI-MS/MS) with a hybrid triple quadrupole-ion trap QTrap 5500 mass spectrometer (AB Sciex, Germany) as described [63, 73] without knowledge of clinical data or treatment groups. Briefly, 2 cycles of liquid-liquid extraction of LPAs were done with 1-buthanol after adding the internal standard LPA17:0 (50 μl sample, 20 μl internal standard in 500 μl 1-butanol). The combined organic phases were dried under a gentle stream of nitrogen at 45 °C and subsequently re-dissolved in 200 μl methanol. A C18 column (Mercury 20 x 2 mm, 3 μm, 100 Å) and precolumn were used for chromatographic separation. A linear gradient was employed at a flow rate of 400 μl/min. Mobile phase A was water with ammonium formate 50 mM and formic acid (100:0.2, v/v) and mobile phase B acetonitrile/formic acid (100:0.1, v/v). The mass spectrometer operated in the negative ion mode and used multiple reaction monitoring (MRM). The mass transitions were m/z 409 → 79 (LPA 16:0), m/z 437 → 153 (LPA 18:0), m/z 431 → 79 (LPA 18:3) and m/z 457 → 153 (LPA 20:4) all with a dwell time of 50 ms. The internal standard method was used for quantification with Analyst Software V1.5 (Applied Biosystems, Germany). The calibration curve was linear over the range of 0.1–500 ng/ml and accuracy was >95%.

### Primary cell culture

The spleens were removed and collected in a sterile wire mesh with 40 μm pore size over a Petri dish half filled with RPMI 1640 media and were gently pressed through the mesh using a 5 ml syringe plunger. The mesh was rinsed over the Petri dish with additional media to remove remaining cells. Disaggregated cells were transferred to a 15 ml Falcon tube and centrifuged at 2,000 rpm for 5 min and washed 2x in RPMI. The cell pellet was resuspended in RPMI, treated with erythrocyte lysis buffer for 10 min at room temperature, followed by centrifugation and resuspension in full medium (RPMI 1640 + 10% FBS + 1% Pen/Strep). Cells were plated in 6-well plates at 3x10^6^/2 ml per well. Growth was stimulated with concanavalin A at 1 μg/ml. Cells were kept in an incubator at 37 °C, 95% humidity and 5% CO2 atmosphere and assays were performed after 2d in culture.

### Enzyme immune assay of autotaxin

Serum samples or crude protein extracts were mixed with a protease inhibitor cocktail (Roche) and subsequently snap frozen in liquid nitrogen and stored at −80 °C until analysis. Proteins from tissue homogenates and primary cells were extracted in PhosphoSafe Buffer (Sigma Germany) containing a protease inhibitor cocktail (Roche) and PMSF 10 μg/ml. The autotaxin ELISAs were used according to the instructions of the manufacturers (R&D systems for human; MyBioScource for mouse). Samples were diluted 1:2 or 1:10, and 50 μl were subjected to the ELISA. Results in tissue are normalized to mg of protein, which was determined with the Bradford assay.

### Autotaxin activity

The lyso-PLD activity of ATX was measured in primary splenocytes of naïve and EAE mice. Cells were incubated for 2 h in 1.1 ml serum free RPMI 1640 medium and then stimulated for another 2 h with PMA 50 ng/ml and ionomycin 500 ng/ml in serum free medium before the autotaxin substrate, C17-LPC was added (100 μM in 1 μl in DMSO). Cell culture supernatants were harvested before adding C17-LPC (baseline) and at 0.5, 1 and 2 h (each sample 100 μl) and cell pellets were collected at 2 h. C17-LPC is converted into the unnatural C17:0-LPA, which was analysed in supernatants and crude cell extracts by LC-MS/MS as described above. LPA18:3 was used as internal standard.

### LPA receptor activation with different LPAs

To study the activation profile of heterologously expressed LPA receptors, COS-1 cells were seeded in 96-well plates at a density of 50.000 cells/well. Next day, they were co-transfected with plasmids containing cDNA for a calcium-sensitive bioluminescent fusion protein between aequorin and GFP [3] and plasmids containing the indicated receptor cDNA or control (empty vector, mock) together with the promiscuous Gα15 [42] at a concentration of 50 ng/well by using FuGENE 6 reagent (Promega) according to manufacturer’s instructions. Forty-eight hours later, cells were loaded with 5 μM coelenterazine *h* (Invitrogen) in HBSS buffer containing 1.8 mM CaCl_2_ and 10 mM glucose for 2 h at 37 °C. Measurements were performed by using a luminometric plate reader (Flexstation 3) for 100 s following ligand stimulation. The area under each calcium transient was calculated by using SoftMaxPro software and expressed as area under the curve (AUC). The following lipids were used for stimulation: 1-Palmitoyl-LPA (LPA16:0), 1-oleoyl-LPA (LPA18:1), 1-stearoyl-LPA (LPA18:0), 1-lineoyl-LPA (LPA18:2), 1-arachidonoyl-LPA (LPA20:4). LPA16:0 and 18:1 were from Cayman, the others from AvantiPolar Lipids.

### FACS analysis

Single cell suspensions were prepared from the spleen, lymph nodes and the lumbar spinal cord. Tissues were rapidly dissected, treated with lysis buffer (DMEM/Accutase (PAA) 1:1, collagenase (3 mg/ml, Sigma), DNAse I (1U/ml, Promega)) for 45 min at 37 °C and followed by mechanical disruption, which was done by forcing the tissue through a nylon mesh with 70 μm pore size (Cell Strainer, BD). For FACS analysis of circulating cells, K+ EDTA blood was used. Blood samples or cell suspensions (100 μl) were mixed with 100 μl HEPES buffer (20 mM HEPES) and 1 ml erythrocyte lysis buffer for 10 min at room temperature and CD16/32 blocking antibody (Fcγ RII/III receptor blocker, BD) for 15 min on ice. Cells were incubated for 20 min at room temperature in staining buffer with the respective fluorochrome labeled antibodies (Additional file 1: Table S1) and were then counted with a flow cytometer (BD FACS Canto II). FACS scans were analyzed with FlowJo 10.08. The controls were FITC, PE, or APC-conjugated rat IgG.

### Quantitative RT-PCR analysis of LPA receptors

Total RNA was extracted from homogenized tissue according to the protocol provided in the RNAeasy tissue Mini Kit (Qiagen, Hilden, Germany), and reverse transcribed using poly-dT as a primer to obtain cDNA fragments. QRT-PCR was performed on an ABI prism 7700 TaqMan thermal cycler (Applied Biosystems, Germany) using the SybrGreen detection system with primer sets and probes designed on the TaqMan software (Additional file 2: Table S2). Amplification was achieved at 59 °C for 40 cycles and confirmed with gel electrophoresis. Transcript regulation relative to the housekeeping gene, Gapdh was determined using the relative standard curve method according to the manufacturer’s instructions (Applied Biosystems).

### Immunofluorescence analysis of immune cell infiltrates in the spinal cord

Mice were terminally anaesthetized with isoflurane and cardially perfused with cold 0.9% NaCl, followed by 4% paraformaldehyde (PFA) in 1x PBS for fixation. Spinal cord and brain were excised, postfixed in 4% PFA for 2 h, cryoprotected overnight in 20% sucrose at 4 °C, embedded in tissue molds in cryomedium and cut on a cryotome (12 μm). Immunofluorescent stainings were done as described [49] (antibodies in Additional file 1: Table S1).

Tiled images of individual sections were captured with equal filter and acquisition parameters to assure comparability independent of genotype or treatment and subsequently stitched to cover the complete ventral horn of the spinal cord. Iba1+ and CD11b + microglia/macrophages in the gray matter and white matter were quantified using the particle counter plugin of FIJI ImageJ. After 8bit conversion, the background was subtracted and the intensity threshold set using the IJ_IsoData algorithm implemented in FIJI. For the particle counter, size inclusions were set to 5–575 μm^2^. Average particles sizes were 20 μm^2^ and 28 μm^2^ for Iba1+ and CD11b + particles, respectively. Three or more non-overlapping images of 3 mice were analyzed per group.

### Statistics

Data are presented as scatter plots with mean ± standard deviation or box plots where the box is the interquartile range and the whiskers show minimum and maximum. Data were analyzed with SPSS 23 and GraphPad Prism 6.0. LPA concentrations were compared between groups using analyses of variance (ANOVA), t-tests or Mann-Whitney U tests according to the data structure and distribution. Trends were analyzed using the Jonckheere-Terpstra trend test. Further analyses consisted of correlation (Spearman’s ρ) and χ^2^ statistics. To assess differences of all LPAs simultaneously, total concentrations were transformed to z-scores according to the equation z = X - μ/σ, where X is the individual value, μ the mean and σ the standard deviation, followed by two-way ANOVA with the within subject factor “LPA” and the between subjects factor “group”, i.e. healthy versus MS in patients and naïve, EAE, EAE-fingolimod, EAE-NTZ in mice. Time courses for LPAs were submitted to analysis of variance for repeated measurements (rm-ANOVA). The within subject factor was ‘time’. Gender was introduced as a between-subject factor and age as covariate. In the case of significant results in the ANOVA, groups were mutually compared with t-tests. The alpha level was set at 0.05 for all comparisons and corrected for multiple testing according to the procedures of Dunnett or Bonferroni.

## Results

### Reduction of serum LPAs in MS patients and EAE mice

**Fig. 1:**
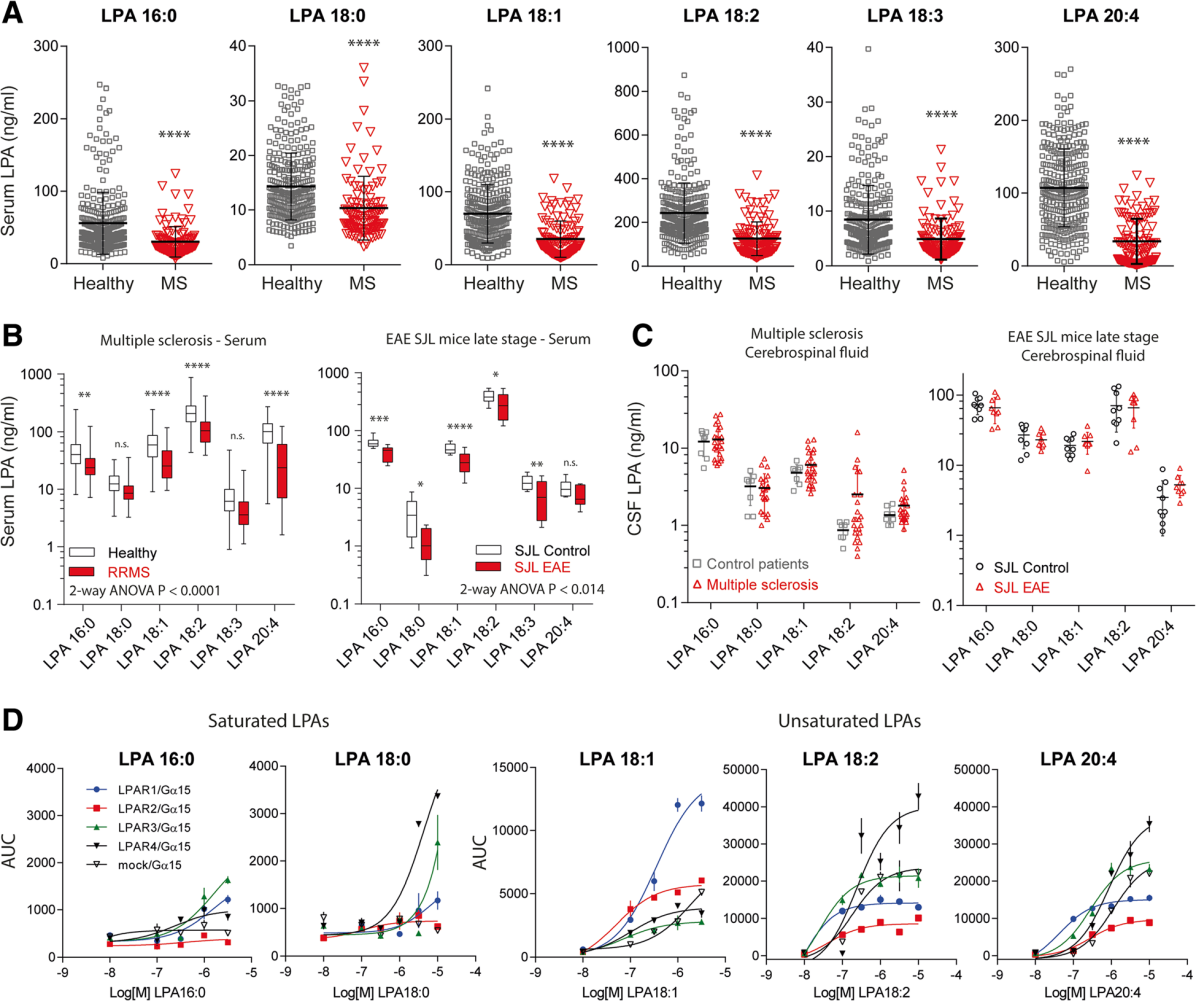
Serum lysophosphatidic acids in MS patients and EAE mice and LPAR preferences. **a** Scatter plots with mean and SD showing the concentrations of lysophosphatidic acids (LPA), LPA16:0, 18:0, 18:1, 18:2, 18:3 and 20:4 in serum samples of 102 patients with multiple sclerosis (demographic data in Table 1) and 301 healthy control. LPAs were analyzed by LC-MS/MS. Data were compared with unpaired, two-sided Student’s t-tests. **b** Box plots showing a comparison of LPA regulations in MS patients with RRMS (*n* = 97) and SJL-EAE mice (*n* = 10). The box represents the interquartile range, the whiskers show minimum to maximum, the line is the median. Data were compared with a 2-way ANOVA, followed by comparisons for each LPA using an adjustment of alpha according to Šidák. **c** Scatter plots showing LPA concentrations in cerebrospinal fluid (CSF) in 24 MS patients versus 8 patients with other neurological diseases and in EAE versus control mice (*n* = 9 per group). EAE was induced in female SJL/J mice with a standard PLP/PTX immunization protocol and clinical scores were monitored. Serum and CSF samples were taken 35 days after immunization, i.e. at the end of the second peak. Control samples were from female, age matched SJL/J mice, injected with CFA without PLP. Data were compared with two-way ANOVA (factors “LPA” and “group”; followed by comparisons for each LPA using an adjustment of alpha according to Šidák). **d** Analysis of LPA receptor preferences of unsaturated and saturated LPAs of different chain length analyzed in COS cells with heterologous expression of LPAR1, 2, 3 or 4 along with the alpha subunit of the promiscuous G-protein G15 (G-alpha15). Data show the mean and s.e.m of 3 replicate analyses. Mock-Gα15 transduced cells were used as controls. For all panels asterisks indicate significant differences between groups, and show adjusted *P* values, *P < 0.05, **P < 0.01, ***P < 0.001; ****P < 0.0001

Concentrations of LPAs with different chain lengths and phosphorylation (LPA16:0, 18:0, 18:2, 18:3 and 20:4) were obtained in the serum of 102 MS patients and 301 healthy controls with equal distribution of sexes (χ^2^ test: *p* = 0.410). All LPAs were significantly reduced in MS patients (two tailed, unpaired Student’s t-tests *P* < 0.0001 for each LPA; Fig. 1a). Similar results were obtained with two-way ANOVA using “LPA” by “group” (for “group”: F (1, 356) = 81.83, *P* < 0.0001; for the interaction “LPA x group” F (5, 1780) = 58.23; *P* < 0.0001) and subsequent Bonferroni posthoc analyses. As healthy subjects were younger than MS patients (Demographic Table 1) the analysis was repeated for subsets of gender and age matched patients and controls > 30 years. The subgroups encompassed 65 MS patients and 36 controls aged 41.0 ± 7.6 and 38.5 ± 7.7 years, respectively (t-test for age: *p* = 0.119). Still, all LPAs were significantly reduced in MS patients (LPA16:0 *P* = 0.015, 18:0 *P* = 0.020, 18:1 *P* <0.0001, 18:2 *P* = 0.002, 18:3 *P* = 0.003, 20:4 *P* < 0.0001). Linear regression analyses did not show a significant association of the sum of all LPAs with age or body mass index (BMI) in healthy controls (correlation coefficients for age: -3.643, *P* = 0.141, BMI −6.680, *P* = 0.181) or age in MS patients (R^2^ = 2.435, *P* = 0.115). BMI data of MS patients were not available.

The LPA pattern of MS patients was compared with that of SJL/J mice in the EAE model of multiple sclerosis (Fig. 1b). Blood was sampled during the second interval 35 days after immunization, where no signs of the disease were detectable. This agrees with majority of patients who mostly had a stable RRMS. Similar to MS patients, EAE mice had significantly reduced LPA serum concentrations compared with control SJL/J mice (2-way ANOVA for “group” F (1, 18) = 7.412, *P* = 0.0140, for interaction “group x LPA” F (5, 90) = 4.667, *P* = 0.0008).

The LPA concentrations in cerebrospinal fluid (CSF) of MS patients did not differ from those of patients with other neurological diseases (Fig. 1c). The demographics of these patients have been described previously [48]. Again, mice were similar to MS patients (Fig. 1c).

### LPAR preferences of different LPAs

**Fig. 2:**
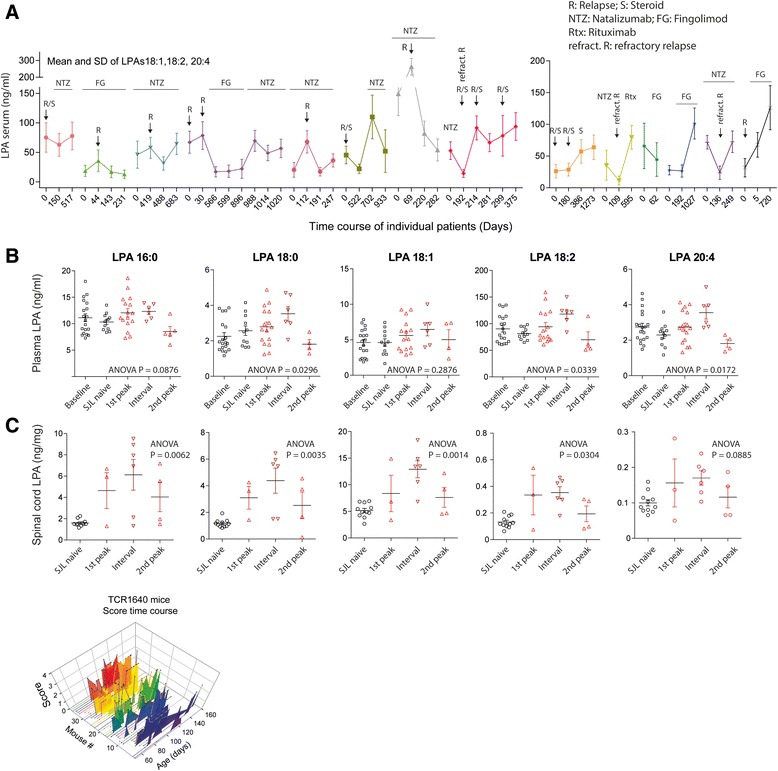
Time courses of LPAs in individual MS patients and in TCR^1640^ mice with spontaneous EAE. **a** LPA serum concentration time courses in RRMS patients. The data show the mean and SD of the unsaturated LPAs 18:1, 18:2, and 20:4. The time of the first sample is referred to as “0”. Patients were observed over several months up to 3 years. R indicates a clinical relapse, R/S is a relapse with steroid treatment, S a steroid pulse for prophylaxis, NTZ and FG show prophylactic treatments with natalizumab and fingolimod, respectively. Rtx = rituximab. The *left panel* shows patients with high LPAs during relapse, the *right panel* shows patients with low LPAs during relapses, which were in part refractory relapses. **b**, **c** Scatter plots with mean and SD showing plasma concentrations and spinal cord tissue concentrations of LPAs in TCR^1640^ mice at different stages of the disease. Data were compared with one-way ANOVA and subsequent post-hoc t-tests employing a Bonferroni correction for multiple testing. *P* < 0.05. The 3D plot (*bottom*) shows the individual time courses of the clinical scores in TCR^1640^ mice. They carry a T-cell receptor (TCR) specific for myelin oligodendrocyte glycoprotein (MOG) peptide 92–106 and develop EAE spontaneously. Each color represents one mouse

**Fig. 3:**
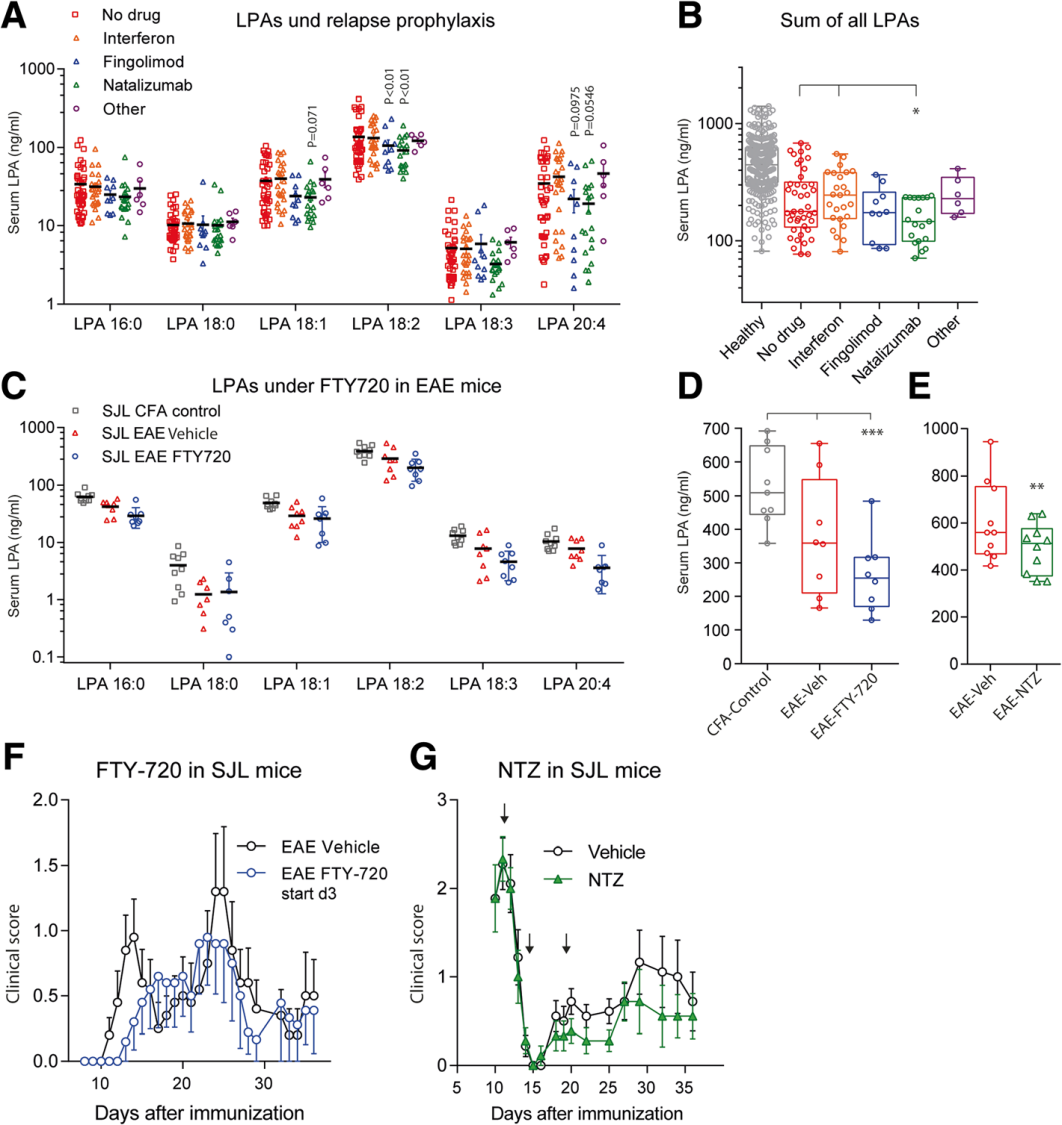
Effects of treatments on LPAs in MS patients and EAE mice. **a**, **b** Box and scatter plots showing serum concentrations of LPAs of different chain length (in **a**) or summed LPAs (in **b**) in MS patients with different prophylactic treatments. The box represents the interquartile range, the whiskers show minimum to maximum, the line is the median, the *dots* show levels in individual patients. **c**, **d**, **e** Box and scatter plots of LPA serum concentration in control mice and in SJL/J mice with EAE, the latter treated either with vehicle, fingolimod or natalizumab. The panel in (**c**) shows the LPAs of different chain lengths, the graphs in (**d)** and (**e**) show summed LPAs. Samples were taken during the second interval, 35 days after immunization. Data were compared with one-way ANOVA and subsequent post-hoc t-tests employing a Bonferroni correction for multiple testing. Asterisks show adjusted *P* values, *P < 0.05, **P < 0.01, ***P < 0.001. **f**, **g** Time course of the clinical EAE scores of SJL/J mice shown in (**c**–**e)** (*n* = 10 per group). Fingolimod treatment with the drinking water (0.5 mg/kg/day) was started 3d after immunization and was continued up to the end. Natalizumab was injected i.p. on d11 (200 μg/mouse), d14 (150 μg/mouse) and d19 (150 μg/mouse)

**Fig. 4:**
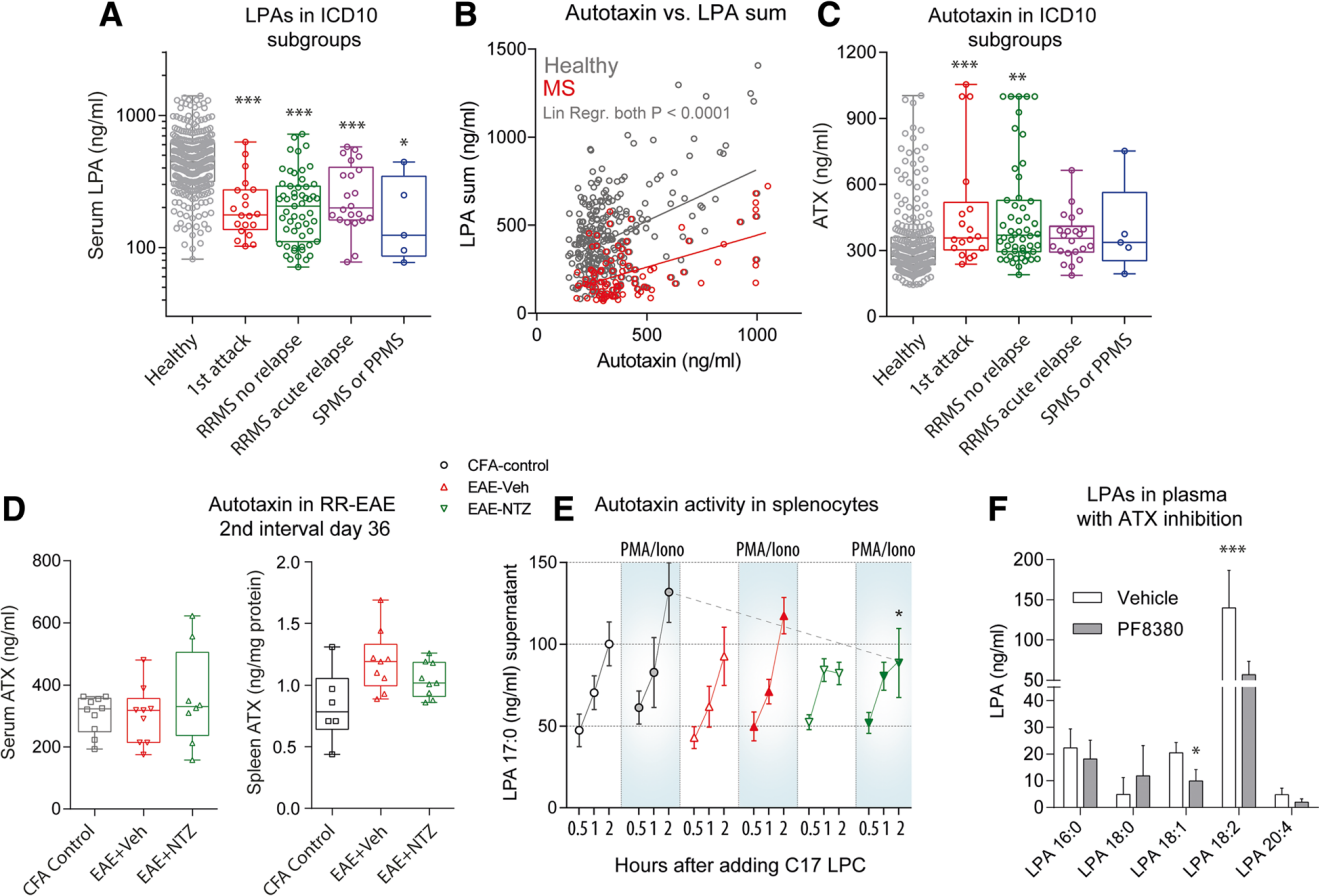
LPAs and autotaxin in ICD10 subgroups of MS patients and EAE mice. **a** Box plots showing the summed concentrations of all types of LPAs in serum of MS patients and healthy controls. The box represents the interquartile range, the whiskers show minimum to maximum, the *line* is the median. Patients were allocated to the groups according to available demographic data. “1^st^ attack” refers to patients with the first clinically manifest course of the disease that led to the diagnosis MS. “RRMS without relapse” are patients that were ‘symptom-free’ at the time of sampling after two or more previous relapses with or without prophylactic treatment. “RRMS with relapse” are patients with acute relapse with or without prophylactic treatment and “SPMS or PPMS” are patients with secondary or primary progressive disease. Data were compared with two-way ANOVA (factors “LPA” and “CD10-group”, posthoc adjustment of alpha according to Šidák. *P < 0.05, **P < 0.01, ***P < 0.001). **b** Scatter plots of autotaxin versus summed LPA concentrations in serum of MS patients and healthy controls and linear regression analyses showing significant associations of LPAs and autotaxin in both groups. **c** In analogy to (**a**), box plots show autotaxin concentrations in ICD10-subgroups. **d** Serum and spleen autotaxin concentrations in EAE and control mice, and in EAE mice treated with natalizumab 35 days after immunization. EAE was induced in female SJL/J mice with a standard PLP/PTX immunization protocol and serum samples were taken 35 days after immunization. Control mice got a CFA injection without PLP. Data were compared with one-way ANOVA. *P* < 0.05. **e** Autotaxin activity in primary splenocytes of EAE (SJL/J 35d after immunization) and control mice as assayed by conversion of C17-lysophosphatidylcholine (LPC) to the unnatural C17 LPA, which was analyzed by LC-MS/MS in cell culture supernatants. Cells were stimulated with PMA/Ionomycin (50/500 ng/ml) or vehicle in serum free medium for 2 h before adding the substrate, C17-LPC (100 μM). 50 μl samples of the supernatant were taken 0.5, 1 and 2 h after adding C17-LPC. Data were compared with 3-way ANOVA with the within subject factors “time” and between subject factors “stimulation” and “treatment”. Effects were significant for “time” *P* < 0.0001, “stimulation” *P* < 0.0001, “treatment” *P* < 0.001 and the respective interactions. Subsequently, corresponding groups were compared with 2-sided, unpaired t-tests employing a Bonferroni correction of alpha. The asterisk indicates a significant treatment effect, *P* = 0.0225. The data (mean ± SD) are results of 6 cultures per group. **f** LPA plasma concentration in naïve mice treated with vehicle or with the autotaxin inhibitor PF8380, which was injected i.p. 30 min before plasma sampling. Data were compared with two-way ANOVA (factors “LPA” and “treatment”) and subsequent 2-sided, unpaired t-tests for each LPA individually; *P < 0.05, *** *P* < 0.001

Regression analyses revealed a linear association between LPAs of different chain length in MS patients showing a congruent regulation, but time courses of individual patients and effects of medication suggested some differences between LPAs of different saturation (Figs. 2, 3 and 4). Therefore, we assessed receptor preferences. The analysis revealed differences between unsaturated and saturated LPAs (Fig. 1d). The former were weak agonists of all heterologously expressed LPARs, whereas the latter activated the LPARs in the nM to low μM range. LPA18:1 was most specific, because mock transduced cells did not respond, and it resulted in highest Emax values for LPAR1 and LPAR2. Two artificial control LPAs, in which the fatty acid is linked via an ether bond (octadecyl-LPA and hexadecyl-LPA) had no effects.

### Relapse associated LPA changes in MS patients and spontaneous EAE

To further assess the effect of relapse and medication in MS patients we analyzed time courses of LPAs in a subset of MS patients over a period of several months to years (Fig. 2). The patients participated in clinical efficacy studies of fingolimod (FTY720) or natalizumab (NTZ). Regulations of LPAs were again congruent. The concentrations of the unsaturated LPAs, LPA18:1, 18:2 and 20:4, i.e. the receptor-activating LPAs, (Fig. 1d) were averaged to show the individual time courses. The patients clustered in two groups: in the first, clinical relapses were associated with elevated LPA concentrations, whereas in the second group the opposite was true, i.e. LPAs were particularly low during relapses, which were in part refractory relapses.

The highly variable course of the disease in MS patients is not well mimicked by the immunization-induced EAE model. Therefore, we used the spontaneous EAE model in TCR^1640^ transgenic mice, which spontaneously develop a T- and B-cell dependent EAE to monitor and compare stage-associated LPA alterations in plasma and spinal cord tissue. The time courses of the clinical scores mimic the highly variable human disease (Fig. 2 bottom). We observed a mild increase in plasma LPAs during the first peak and a stronger increase in the first interval that was followed by a decrease in the late stage of the disease (Fig. 2b), the latter similar to the situation in patients. We used plasma instead of serum for this experiment to exclude the impact of platelets. LPA concentrations in plasma are about 5–10 fold lower than in serum. The increase during the 1^st^ interval was associated with a stage-dependent increase in the lumbar spinal cord (Fig. 2c) i.e. in a ‘symptom-free’ period reflecting the resolution of inflammation.

### Effects of treatments on LPA changes

Patients with RRMS normally receive prophylactic treatment to reduce the frequency and number of relapses. Two of the latest available drugs are the sphingosine-1-phosphate analog, fingolimod and the alpha-4 integrin (VLA4) targeting monoclonal antibody, natalizumab. To assess the effect of treatment we compared serum LPAs in medication-subgroups in MS patients and EAE mice and the clinical EAE courses in SJL/J mice receiving these treatments (Fig. 3). LPA concentrations in serum of patients treated with fingolimod or natalizumab were significantly lower than in patients not receiving medication, or receiving beta interferon or other drugs including glatiramer, azathioprine, repeated glucocorticoid cycles or fumarate (two-way ANOVA for factor ‘medication’ F (4, 103) = 3.012468, *P* = 0.0214 and ‘medication x LPA’ F (20, 515) = 2.267692, *P* = 0.0014; posthoc Dunnett results in Fig. 3a, b). The additional LPA lowering effect was also observed in fingolimod or natalizumab treated EAE mice (Fig. 3c–e). Comparison of EAE versus EAE & Fingolimod was highly significant for LPA18:2 and the sum of all LPAs (one-way ANOVA F (2, 22) = 7.995962, *P* = 0.0025, posthoc in Fig. 3d), but the trend was also visible for LPA16:0, 18:1, 18:3 and 20:4 (Jonckheere’s trend tests for these LPAs all *P* < 0.001). Similarly, natalizumab treated mice had significantly lower LPA levels than vehicle treated mice (Fig. 3e). We used NTZ although it has been raised against human Itga4, which has 85% identity with mouse Itga4. NTZ treatment in SJL/J mice, consisting in 3 injections at d11, 14, 19, reduced the clinical scores whereas fingolimod, which is highly active in C57BL6 mice, had only minor effects in SJL/J mice as shown before [13] (Fig. 3 f, g).

### LPAs and autotaxin in ICD10 subgroups of patients

We compared levels of LPAs in disease subgroups according to the clinical ICD10 score (Fig. 4a). In all MS subgroups, serum LPA levels were significantly lower than in healthy controls and subgroups were similar, but patients with acute relapse tended to have higher levels (n.s.) than the other patient groups, which is in line with the individual time courses of patients in cluster-1 (Fig. 2). Extracellular LPAs are mainly produced by autotaxin and we found a linear association of LPAs with autotaxin concentrations both in healthy subjects and MS patients (Fig. 4b). However, ICD10 subgroup associations with serum autotaxin were rather inverse to those of LPAs (Fig. 4c). In RR-EAE, serum autotaxin levels were unaltered during the ‘symptom-free’ second interval (Fig. 4d) but autotaxin levels were increased in the spleen, possibly reflecting local upregulation. Ex vivo, ATX activity in primary splenocytes was similar in control and EAE mice (Fig. 4e), but the maximum stimulation effect of PMA/Ionomycin was significantly reduced in splenocytes from natalizumab treated mice (Fig. 4e). Inhibition of ATX with PF8380 mainly reduced unsaturated LPAs in plasma in naïve mice (Fig. 4 f), again suggesting that saturated and unsaturated LPAs were differentially regulated.

### LPA receptor subtypes in immune cells

**Fig. 5:**
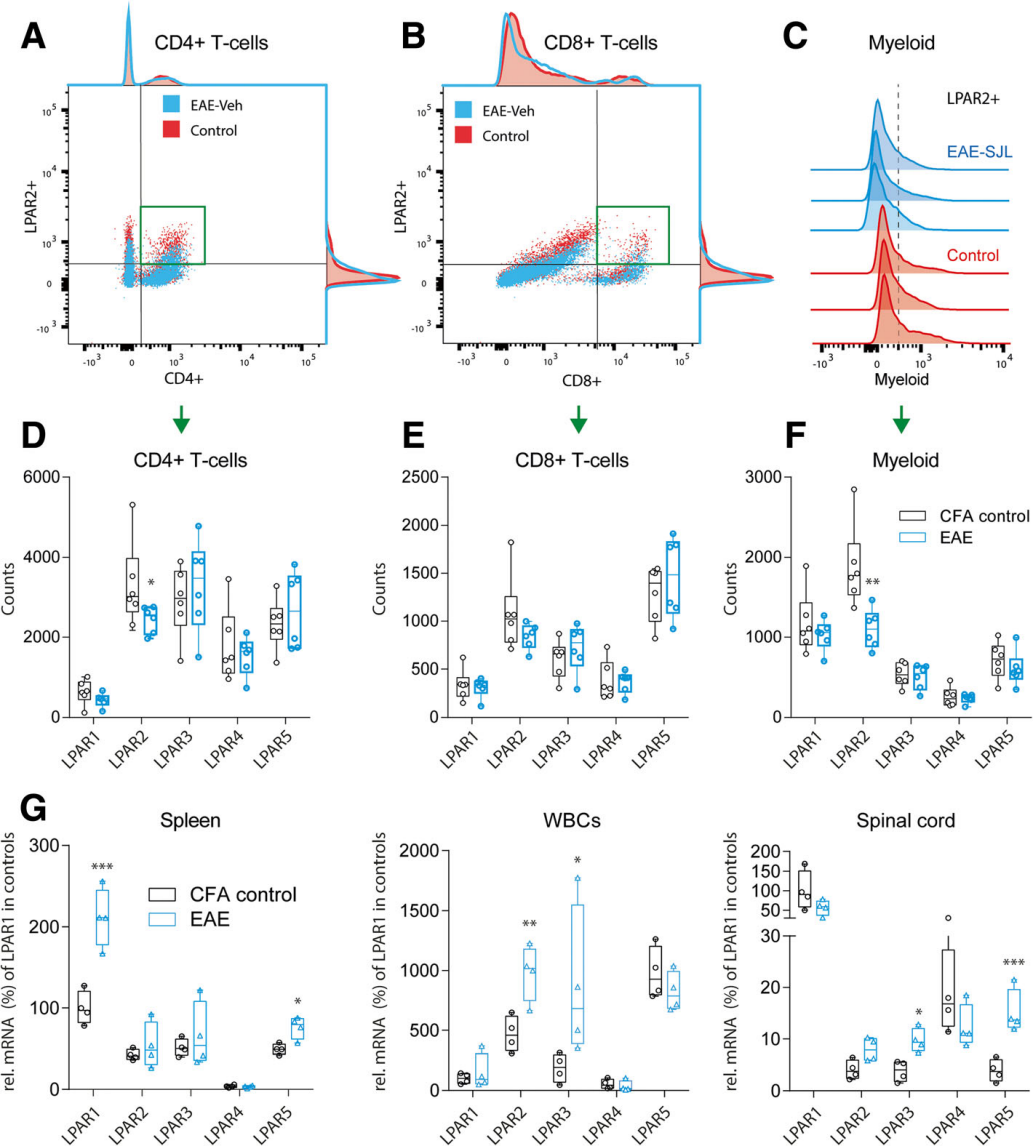
Expression and regulation of LPARs. **a**, **b** FACS analyses of splenic immune cells in control and EAE mice showing exemplary scatter plots of T-helper and T-suppressor cells gated according to CD4+ versus LPAR2 and CD8+ versus LPAR2. The spleens were taken during the second interval in SJL/J mice, 35 days after immunization. Control mice had received an injection of CFA without PLP. The *green* framed area shows the double positive cells, which were used for quantification. **c** Exemplary histograms of myeloid cells gated according to the expression of LPAR2. Each 3 examples of control and EAE mice are shown. **d**, **e**, **f** Box and scatter plots of the total numbers of LPAR positive CD4+ and CD8+ T-cells and of CD11c ∩ F4/80 positive myeloid cells. 100,000 cells were counted of each 6 mice. Boxes show the interquartile range, whiskers minimum to maximum and the *circles* show results of individual mice. The *line* is the median. Data were compared with unpaired 2-tailed t-tests for each LPAR separately. *P < 0.05, **P < 0.01. **g** Box and scatter plots of the mRNA levels of LPAR1, 2, 3, 4 and 5 in the spleen, white blood cells (WBCs) and spinal cord in SJL-EAE mice, 35 days after immunization i.e. after the second peak. The mRNA levels were normalized to the delta Ct level of LPAR1 in control mice, which was set to 100%. Hence, the data show EAE effects and overall differences in the tissue specific LPAR expression patterns. Data were compared with unpaired 2-tailed t-tests for each LPAR separately. *P < 0.05, **P < 0.01, *** *P* < 0.001, meaning of the boxes as in (**d**–**f**)

To assess functional implications of LPA alterations, we analyzed their receptors in immune cells in the context of EAE (Fig. 5). FACS analyses of LPARs in combination with markers of T-cell subpopulations revealed a decrease in LPAR2 positive CD4+ T-cells and LPAR2+ myeloid cells in the spleen suggesting reduced homing or increased egress (Fig. 5a–f). To assess the traffic of these cells we analyzed LPAR mRNAs in spleen, white blood cells and spinal cord (Fig. 5g). Indeed, LPAR2 mRNA strongly increased in WBCs and spinal cord in EAE mice, and LPAR3 was similarly regulated, suggesting that LPAR2 and 3 positive immune cells entered the spinal cord. LPAR1 strongly increased in the spleen in line with its high expression in dendritic cells [54]. LPAR5 increased locally in the spleen and strongly in the spinal cord but not in the blood suggesting that the increase in the spinal cord was not due to invasion but rather local cell proliferation, likely glia. According to a previous study of FACS sorted cells of the lymph nodes [54], dendritic cells primarily express LPAR1 and 3, B-cells and T-cells LPAR2 and 5, and neurons mainly carry LPAR2 and LPAR5 [63, 69]. In terms of glia, previous studies revealed expression of LPAR1 in oligodendrocytes [66] where it was essential for proper myelination [23], cortical development [17] and normal proliferation, maturation and differentiation of neuronal precursors [36]. LPAR1 was also important for Schwann cell survival and migration [1, 65] and LPA treated astrocytes induced axonal outgrowth [51, 52].

### EAE in LPAR2 deficient mice and therapeutic effects of LPAR2 agonist

**Fig. 6:**
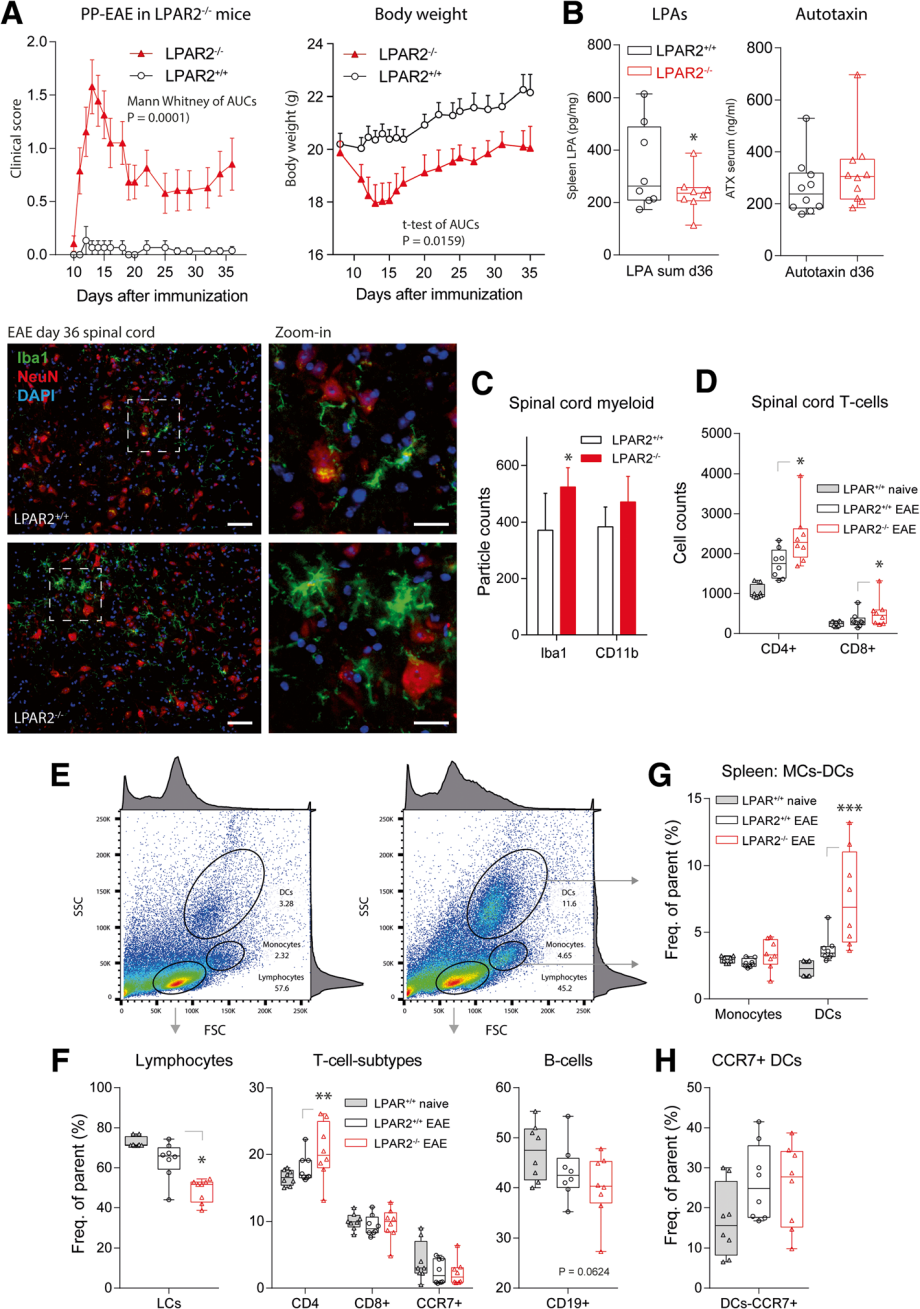
EAE in LPAR2 deficient mice. **a** Time course of the clinical EAE scores and body weight of LPAR2^−/−^ versus LPAR2^+/+^ mice in the MOG-induced primary progressive EAE model (*n* = 19 for LPAR2^−/−^ and *n* = 15 for LPAR2^+/+^). LPAR2 mice are on a mixed C57BL6xSv129 genetic background (Sv129 EAE unresponsive), so that the wild type LPAR2^+/+^ mice have very mild signs of EAE. AUCs of the scores and body weights were compared with Mann Whitney U test and 2-tailed unpaired t-test, respectively. *P* < 0.05. **b** Box plot of the sum of all LPAs in the spleen and autotaxin in serum 36 days after immunization. The boxes show the interquartile range, the *line* is the median, the whiskers show minimum to maximum and the *dots* are results of individual mice. The asterisk shows a statistically significant difference between groups, 2-tailed unpaired t-test, *P* < 0.05. **c** Exemplary immunofluorescence images taken from the lumbar spinal cord of LPAR2^−/−^ and LPAR2^+/+^ mice. The images show the myeloid marker, Iba-1 with a neuronal counterstain of NeuN and DAPI for labeling of nuclei. The dashed rectangles show the areas taken for zoom-in images. Scale bar 50 μm and 25 μm in zoom-in images. Cell counts for Iba-1 or CD11b positive cells were obtained with FIJI ImageJ for *n* = 6–9 images of 3–4 mice per group and compared with a 2-way ANOVA for “marker” versus “genotype”. The counts differed significantly for Iba-1 between genotypes (*P < 0.05), and the morphology of large rhomboid microglia in LPAR2^−/−^ mice suggested a stronger activation. **d** FACS analysis of the number of CD4+ and CD8+ T-cells in the lumbar spinal cord in LPAR2^−/−^ and LPAR2^+/+^ mice 36 days after immunization. The asterisks show significant differences between LPAR2^−/−^ and LPAR2^+/+^ mice (2-way ANOVA and subsequent t-tests according to Dunnett for each cell population, *P* < 0.05). **e** Exemplary forward (FSC) versus sideward (SSC) scatter plots of the immune cells in the spleen in LPAR2^−/−^ and LPAR2^+/+^ mice 36 days after immunization. Lymphocytes, monocytes and DCs (myeloid cells) were identified by size and granularity. **f** Box plots showing the quantification of lymphocytes, T-cell subtypes and B-cells in the spleen in LPAR2^−/−^ and LPAR2^+/+^ mice 36 days after immunization (meaning of boxes as in **b**). Asterisks denote significant difference between groups (* *P* < 0.05, *** *P* < 0.001, one-way ANOVAs with subsequent t-tests versus EAE-LPAR2^+/+^ mice according to Dunnett). **g**, **h** Box plots showing the quantification of myeloid cells in the spleen in LPAR2^−/−^ and LPAR2^+/+^ mice 36 days after immunization. Expression of the homing receptor CCR7 by dendritic cells is a marker of their activation (meaning of boxes as in **b**, statistics as in **f**)

Because we observed the most interesting effects on LPAR2+ populations, we opted for this candidate to assess potential functions and studied the clinical course of the EAE disease in *Lpar2* deficient mice [12]. LPAR2^−/−^ and LPAR2^+/+^ control mice have a mixed C57BL6/Sv129 background. Sv129 is a non-permissive strain, so that LPAR2^+/+^ control mice show very mild clinical signs of EAE. In contrast LPAR2^−/−^ developed a strong primary progressive EAE reaching scores of 2.5–3 accompanied by a loss of the body weight (Fig. 6a). The LPA levels in the spleen were significantly reduced in LPAR2^−/−^ mice 36 days after immunization whereas serum autotaxin levels were not affected at this time point (Fig. 6b). The differences in the clinical scores were reflected by stronger activation of microglia in the grey matter of the spinal cord (Fig. 6c) and stronger infiltration with T-cells in LPAR2^−/−^ mice (Fig. 6d). FACS analyses of immune cells in the spleen revealed a relative reduction of lymphocytes and B-cells with a higher proportion of CD4+ T-cells in LPAR2^−/−^ mice (Fig. 6e, f). LPAR2 deficiency was also associated with an increase in dendritic cells in the spleen (Fig. 6g). Dendritic cells expressing the homing receptor CCR7+ were increased in both EAE groups (Fig. 6h). Hence, if LPAR2 was not present, more T-cells trafficked from the spleen to the spinal cord and we infer that LPAR2 deficiency leads to a defect in lymphocyte homing.

**Fig. 7:**
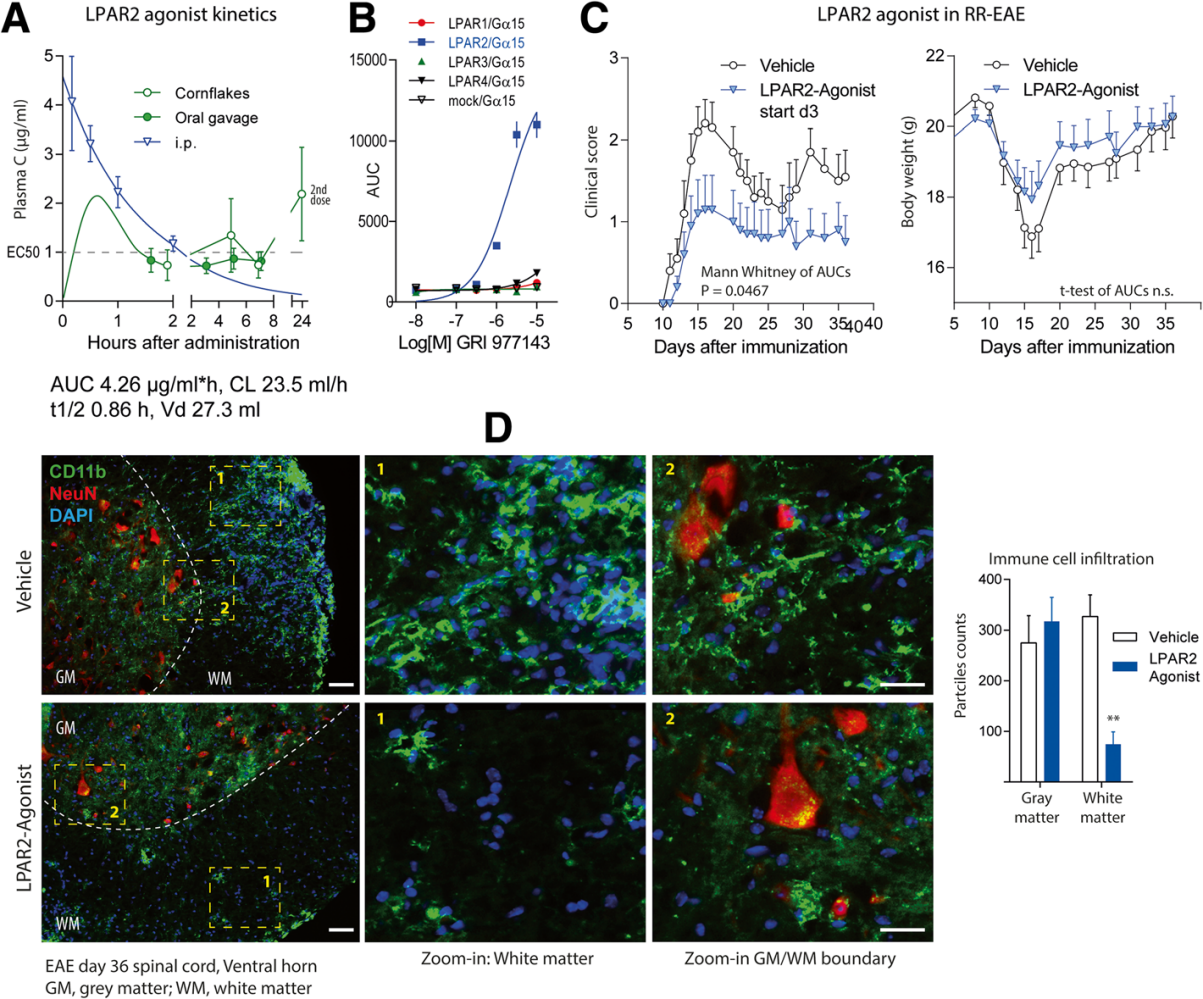
EAE in RR-EAE SJL/J mice treated with an LPAR2 agonist. **a** Plasma concentration time courses and basic pharmacokinetics of the LPAR2 agonist GRI 977143 in SJL/J mice after intraperitoneal injection (i.p.), oral gavage or administration with soaked cornflakes (*n* = 5–6 per group, i.e. per way of administration). Basic PK parameters were calculated from i.p. data using non-linear, 1-phase decay curve fitting. **b** Analysis of LPA receptor activation in COS-1 cells transfected with human LPAR1, 2, 3 or 4 along with the promiscuous G-protein alpha subunit (Gα15). Cells were stimulated with the LPAR2 agonist GRI 977143 to confirm LPAR2 specificity. The EC50 was in agreement with the reported EC50 of about 1–3 μM. Data show the mean and sem of 3 replicate analyses. Mock- Gα15 transduced cells were used as controls. **c** Time courses of clinical EAE scores and body weights of SJL/J mice treated with the LPAR2 agonist GRI 977143 or vehicle perorally starting 3d after immunization (*n* = 10 per group). AUCs of the scores and body weights were compared with Mann Whitney U test or 2-tailed, unpaired t-test respectively, *P* < 0.05. **d** Immunofluorescent analysis of myeloid cell infiltration (CD11b *green*) into the *white* matter of the ventral horn in mice treated with vehicle or the LPAR2 agonist, GRI 977143, 36 days after immunization. Neurons were counterstained with NeuN (*red*) and nuclei are stained with DAP (*blue*). The *white* dashed lines show the boundary between the *gray* and *white* matter. The dashed *yellow* rectangles show the areas taken for zoom-in images. Scale bars 50 μm for overview, 25 μm for zoom-in images. Cells were quantified with FIJI ImageJ using images of 4 mice per group. CD11b positive particles were significantly reduced in the *white* matter of GRI 977143 treated mice, 2-way ANOVA for “site” by “genotype”, posthoc according to Šidák, **P < 0.01 for “genotype”

To assess potential therapeutic implications, we treated RR-EAE SJL/J mice with the LPAR2 agonist, GRI 977143 (100 μg/mouse/d p.o.) starting 3 days after immunization for up to 35d (Fig. 7). Analysis of plasma concentrations (Fig. 7a) revealed rapid absorption after i.p. administration with a Tmax of 10 min, reaching plasma concentrations well above the EC50 of about 1 μM, which were maintained for 2–3 h. Levels in the range of the EC50 were also reached after oral administration and maintained for 4–6 h. The half-life was 0.85 h. We confirmed the LPAR2 specificity in COS cells with heterologous expression of LPAR1, 2, 3 or 4 (Fig. 7b). LPAR2-agonist treated mice had significantly lower clinical EAE scores throughout the treatment period but with high inter-individual variability (Fig. 7c). The therapeutic efficacy was also evident in terms of the body weight (Fig. 7c) and the infiltration of the white matter of the lumbar spinal cord with myeloid cells (Fig. 7d), which was much reduced in mice receiving the LPAR2-agonist. Hence, the LPAR2 agonist may counterbalance the loss of endogenous LPAs.

## Discussion

The present study revealed a deficit of extracellular peripheral LPAs in MS patients and in EAE mice, whereas spinal cord LPAs increased during the remyelination phase in EAE. The apparent peripheral LPA deficiency was associated with impaired homing of LPAR2+ T-cells to the spleen and intensified clinical signs of EAE in LPAR2^−/−^ mice. Conversely, mice treated with an LPAR2 agonist were partially protected from the disease. Together these loss- and gain-of-function experiments suggest that LPAs have protective functions in MS and EAE (i) by increasing lymphocyte homing and (ii) possibly, by promoting remyelination. The latter idea is suggested by previous studies showing that LPAR1 is highly expressed by oligodendrocytes [66] and essentially contributes to myelination [23], Schwann cell migration [1] and proper cortical development [17, 20]. Hence, individual time courses of LPAs may point to changes in disease activity.

It is presently not clear whether different LPAs have specific functions, prefer specific LPARs or are preferentially produced by autotaxin or phospholipase A in the settings examined here. Mostly, we found congruent regulations of all LPAs with linear relationships between LPAs, but the in vitro studies in COS cells suggest that unsaturated LPAs are the major LPAR activators. Indeed, the disease-course associated fluctuations of the unsaturated LPAs matched the individual activity of the disease, were affected by natalizumab and fingolimod, and were exclusively reduced by autotaxin inhibition. While our data mainly suggest protective functions, LPAs may also trigger pro-inflammatory cellular responses [39, 41], depending on the receptor and the source. For example, platelet-derived LPAs trigger the degranulation of neutrophils [58] that contribute to MS pathology [30] and a recent study observed a reduction of EAE manifestations with an autotaxin inhibitor that blocks its catalytic phosphodiesterase activity [55]. The individual LPA time courses of our patients did not give a clear answer, because patients clustered in 2 groups, one with high levels of LPAs during relapse, the other with particularly low levels, which were in part refractory relapses non-responding to treatments. Glucocorticoids, which are frequently administered during relapses may interfere with the ‘natural’ LPA course, because they may inhibit autotaxin [53] and sPLA2 [31], the latter actually being unfavorable for the immune regulatory functions of regulatory T-cells (T-regs) [64]. The observed regulation of autotaxin points to a functional dichotomy but the functions of ATX for myelination [14, 19], although likely mediated by its MORPHO domain and not affected by inhibitors of its phosphodiesterase activity, rather discourages targeting ATX for treatment of MS, supported by our data.

The MS- and EAE-associated reductions of LPAs were further intensified under treatment with fingolimod and natalizumab, affecting mainly unsaturated LPAs. Notably, these effects were seen in MS patients and in the EAE model arguing for genuine effects of natalizumab and fingolimod rather than putative effects caused by higher disease-activity, which is important because fingolimod and natalizumab are highly potent agents restricted to an escalation medication. The effect of fingolimod may be due to autotaxin inhibition [46]. The effects of natalizumab are possibly due to interference with the binding of autotaxin to integrin, which is required for targeting the enzyme to cell surfaces to get access to its substrate, phosphatidylcholine (LPC) [26], and indeed, the maximum stimulated activity of autotaxin was reduced in splenocytes of natalizumab-treated EAE mice. We used natalizumab in the EAE experiments to match the human data. NTZ was raised against human Itga4, which is 85% identical with the mouse protein, and binds human and mouse Itga4 differently [68] but prevents T-cell BBB penetration in EAE mice in vivo [11] and effectively reduced the EAE scores in our mice.

In spontaneous EAE mice, the peripheral LPA loss occurred during a late stage of the disease like in the majority of patients whereas the ‘symptom-free’ interval was associated with LPA increases in the lumbar spinal cord. Hence, the central LPA peak paralleled the resolution of inflammation and time of remyelination. One may interpret this local increase as a sign of protective central effects of LPAs through their receptors on myelinating cells [1, 23, 66] as well as neural stem cells [28, 34, 65, 66]. Consistently, autotaxin is increasingly released at the onset of myelination [19] and maintains functions of oligodendrocytes [22], and secreted PLA2 (sPLA2), which is an extracellular source of LPAs, protects oligodendrocytes from cell death [59], further consistent with LPA’s survival effects on Schwann cells [65]. MS and EAE evoked reductions of LPAs therefore suggest unfavorable adaptations, and medication-evoked further reductions may be side effects compromising LPA-regulated immune cell traffic, but this apparently does not curtail the proven efficacy of fingolimod or natalizumab in human MS [9, 27] but possibly may increase the risk of side effects.

Fingolimod is supposed to act through a retention of immune cells in the lymphoid organs suggesting that bioactive lipids are key regulators of the interplay of homing and egress [8]. The entry of naive lymphocytes into the lymph nodes occurs at high endothelial venules, requiring attachment and extravasation into the tissue [15, 32]. Although the exact mechanisms of LPA mediated transmigrations are under debate [32, 38] it is conceivable that a permanent reduction of LPAs interferes with the migrating properties [2, 70]. It is presently unknown whether LPAs act on T-cells, endothelial cells, or both, and different models have been proposed. Locally generated LPAs may facilitate T-cell exit from the circulation and entry into lymph nodes via T-cell LPARs [70], mainly LPAR2 and LPAR5 [54]. The ATX/LPA signaling may also regulate T-cell homing indirectly by acting on LPARs of endothelial cells [2], which express LPAR1 and LPAR4 [35]. In addition, LPAs modify the cytoskeleton of the endothelial cells necessary to modify their shape and motility, and ATX inhibitors can block lymphocyte extravasation [2, 32]. Regardless of the exact mechanisms involved, the possibility that LPAs promotes T-cell homing and recirculation through lymphoid organs is an exciting new direction and complements the well-recognized role of sphingosine 1-phosphate (S1P) in lymphocyte egress [16]. Hence, LPAs and S1P may act as functional antagonists in this process. The observed decrease of LPAR2 positive T-cells and myeloid cells in the spleen, increase of its mRNA in circulating WBCs and in the lumbar spinal cord, strongly suggest that LPAR2 is one of the crucial receptors. In line with this idea, LPAR2 deficient mice developed stronger EAE than controls and treatment with the LPAR2 agonist reduced clinical signs of EAE, albeit with high variability. In addition to immune effects, both LPA and S1P receptors are widely expressed in the CNS where they are likely to have a range of direct CNS activities relevant to both EAE and MS, as demonstrated by a requirement for astrocyte S1P1 for fingolimod activity in EAE [5, 24] and LPAR1 for myelination [23]. Moreover, promiscuity of lipid ligands as shown by endocannabinoid metabolites acting through LPA receptors [7] adds further potential to explain variation. Cell-type specific LPAR-deficient mice will help to dissect out specific LPAR functions in the future, and possibly dual agonists may have less variable efficacy.

## Conclusion

In summary, we show a reduction of LPAs in serum in patients with multiple sclerosis, individual relapse dependent re-raises or further downregulations, and further reductions under treatment with fingolimod and natalizumab. The alterations are also evident in mice with relapsing-remitting EAE and spontaneous EAE and the LPA loss was associated with reduced lymphocyte homing of LPAR2 positive T-cells. Finally, complete deficiency of LPAR2 aggravated EAE whereas an LPAR2-agonist treatment attenuated the disease. Together, the data suggest that functional deficits of LPA-LPAR2 signaling contribute to the pathophysiology of multiple sclerosis and possibly might be targeted by specific treatments.

## Additional files

Additional file 1: Table S1. Lists of antibodies. (DOC 36 kb)

Additional file 2: Tables S2. Lists of primers. (DOC 29 kb)
