# Supplementary material for: Dysregulation of lysophosphatidic acids in multiple sclerosis and autoimmune encephalomyelitis
Source: Acta Neuropathol Commun. 2017 Jun 2;5:42. doi: 10.1186/s40478-017-0446-4 (PMC5457661; doi:10.1186/s40478-017-0446-4)
Supplement: Supplementary file 1 — Lists of antibodies. (DOC 36 kb) [file 40478_2017_446_MOESM1_ESM.doc]

**Additional file 1: Table S1: Lists of antibodies**

| **Antibody** | **Conjugate** | **Company** | **Concentration** | **Use** | **Marker for** |
| --- | --- | --- | --- | --- | --- |
| **CD4** | eFluor500 | BD | 0.2 mg/ml | FACS | T-helper cells |
| **CD8** | PerCPeFluor710 | eBioscience | 0.2 mg/ml | FACS | T-suppressor cells |
| **CD19** | PE | eBioscience | 0.2 mg/ml | FACS | B-cells |
| **CCR7** | APC | eBioscience | 0.2 mg/ml | FACS | Dendritic cells, activated |
| **CD11b** | eFluor450 | eBioscience | 0.1 mg/ml | Immunofluorescence | Monocytes, macrophages, microglia |
| **LPAR1** | FITC | Bioss | 1 mg/ml | FACS |  |
| **LPAR2** | FITC | Bioss | 1 mg/ml | FACS |  |
| **LPAR3** | FITC | Biorbyt | 0.5 mg/ml | FACS |  |
| **LPAR4** | FITC | Bioss | 1 mg/ml | FACS |  |
| **LPAR5** | FITC | Bioss | 1 mg/ml | FACS |  |
| **Iba1** |  | SantaCruz | 0.2 mg/ml | Immunofluorescence | Macrophages, microglia |
| **NeuN** |  | Merck Millipore | 1 mg/ml | Immunofluorescence | Neuronal nuclei |
