# Supplementary material for: Dysregulation of lysophosphatidic acids in multiple sclerosis and autoimmune encephalomyelitis
Source: Acta Neuropathol Commun. 2017 Jun 2;5:42. doi: 10.1186/s40478-017-0446-4 (PMC5457661; doi:10.1186/s40478-017-0446-4)
Supplement: Supplementary file 2 — Lists of primers. (DOC 29 kb) [file 40478_2017_446_MOESM2_ESM.doc]

Suppl. Tables 2: Lists of primers

| **Gene** | **Forward** | **Reverse** |
| --- | --- | --- |
| LPAR1 | CCTCTTCATCGCCCCAAACT | TTCATGGCTGTGAACTGGGG |
| LPAR2 | CATTCTGGGGGCATTTGTGG | TTGACCAGTGAGTTGGCCTC |
| LPAR3 | GTCTTAGGCGCCTTCGTGG | TTGCACGTTACACTGCTTGC |
| LPAR4 | GCGAGTTGCCAGTTTACACG | TTGAGTGCCCAAGAAAGAGTGT |
| LPAR5 | CAAGAAGGTCTCCACTGCTGA | GTGGTAGCCTGGTGGCAATA |
| Gapdh | CCTCGTCCCGTAGACAAAATG | TCTCCACTTTGCACTGCAA |
